# The causal effects of leisure screen time on irritable bowel syndrome risk from a Mendelian randomization study

**DOI:** 10.1038/s41598-023-40153-1

**Published:** 2023-08-14

**Authors:** Liesheng Lu, Changqin Liu, Kunpeng Liu, Chenzhang Shi, Zhongchen Liu, Xun Jiang, Feng Wang

**Affiliations:** 1 Department of General Surgery, Shanghai Tenth People’s Hospital, Tongji University School of Medicine, 301 Middle Yanchang Road, Shanghai, 200072 China; 2 Department of Gastroenterology, Shanghai Tenth People’s Hospital, Tongji University School of Medicine, Shanghai, China; 3 Bengbu First People’s Hospital, Bengbu, China

**Keywords:** Genetics, Gastroenterology, Risk factors

## Abstract

Associations between leisure sedentary behavior (especially leisure screen time, LST) and irritable bowel syndrome (IBS) have been reported, but causality is unclear. Here, the two-sample Mendelian randomization was performed to investigate the causal association between LST and IBS. Two recently published genome-wide association studies (GWASs) including a total of 1,190,502 people from Europe were used as our data source. Inverse variance weighting (OR = 1.120, 95% CI 1.029–1.219) and weighted median (OR = 1.112, 95% CI 1.000–1.236) analyses revealed a causal effect between LST and IBS. There was no evidence of pleiotropy in the sensitive analysis (MR-Egger, p = 0.139). After removing potentially confounding single nucleotide polymorphisms (SNPs), similar results were found using inverse variance weighting (OR = 1.131, 95% CI 1.025–1.248) and weighted median (OR = 1.151, 95% CI 1.020–1.299), as well as in the validation analyses using inverse variance weighting (OR = 1.287, 95% CI 0.996–1.662). This study provided support for a possible causal relationship between leisure screen time and IBS.

## Introduction

As a common disorder of the digestive tract, Irritable bowel syndrome (IBS) causes periods of abdominal pain, bloating, and altered bowel patterns. This functional condition of the lower gastrointestinal tract affects roughly 5–10% of the world's population and has considerable influence on patient's quality of life, the economy and society as a whole^1,2^. Currently, its etiology and pathogenesis are thought to be related to genetics, intestinal motility, infection, and chronic inflammation. The disease's severity may be amplified, however, by other conditions that may coexist with it, such as one's way of life and emotional stress^3^. Consequently, a comprehensive comprehension of IBS risk factors is helpful to discovering its mechanism, given that there is no unambiguously effective treatment for IBS^4^.

Physical inactivity has an important impact on various chronic illnesses, including cardiovascular disease, diabetes, and cancer^5^. Increasing sedentary time coincides with declining physical activity levels, which may constitute an independent danger to public health^6^. Observational studies are finding more and more evidence that extended leisure sedentary behavior, particularly leisure screen time (LST), is related with an higher risk of some cancers and total death^7,8^. However, there is little evidence to support a relationship between LST, a main sedentary activity, and an increased risk of IBS. Furthermore, the effects of residual confounding and/or reverse causality limit the usefulness of observational study results to draw causal conclusions.

The Mendelian Randomization (MR) strategy substitutes genetic variants for a risk factor in an instrumental variable analysis, because they are randomly assigned during meiosis and are thus unaffected by the possible confounders that bias observational studies^9^. In the current work, we used GWAS summary data to conduct an MR analysis to better understand the possible causal relationship between LST and IBS risk.

## Methods

### Mendelian randomization and assumptions

**Figure 1:**
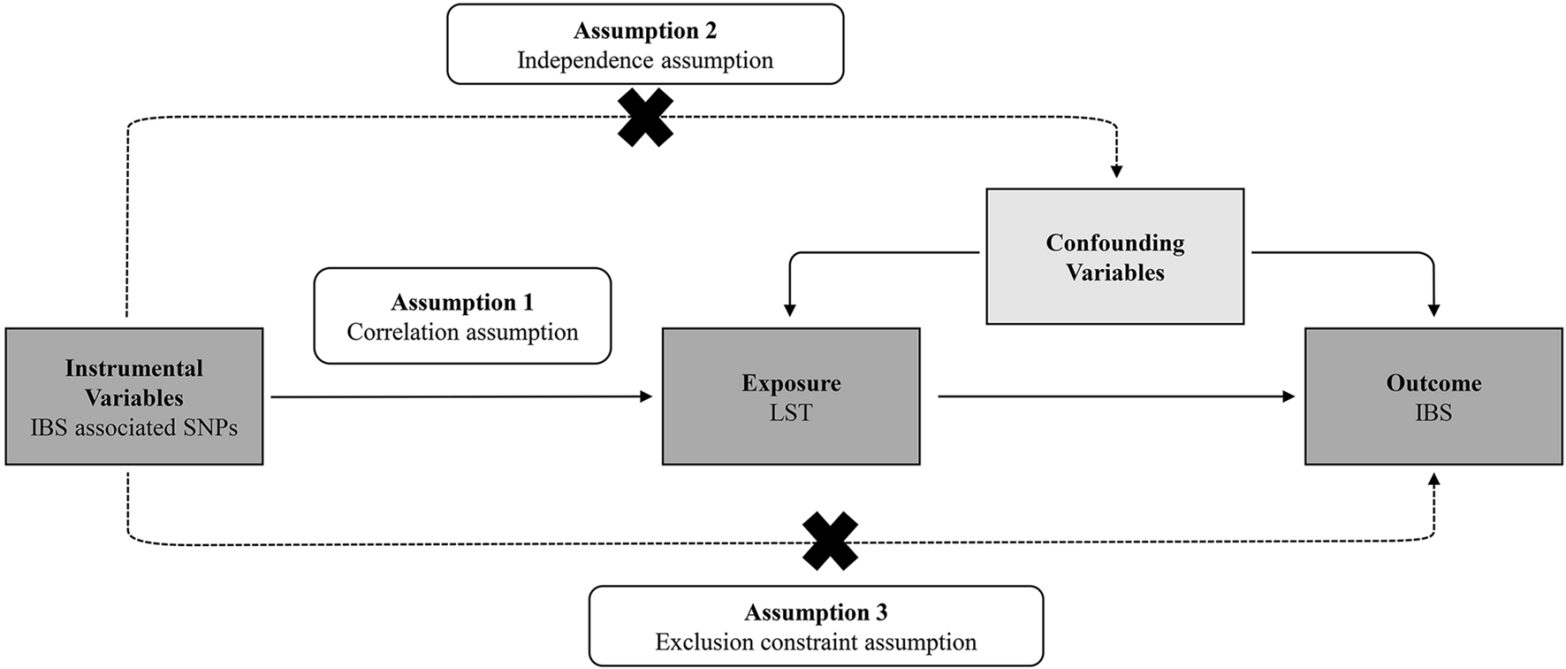
Schematic representation of Mendelian randomization analysis. *IBS* irritable bowel syndrome, *LST* leisure screen time, *SNPs* single-nucleotide polymorphisms.

In an MR investigation, genetic variations or SNPs serve as the independent variables in an instrumental variable analysis (IVs). As valid IVs, the SNPs must satisfy the three core assumptions of MR: (1) the SNPs are strongly associated with the exposure (leisure screen time); (2) Each SNP is independent of confounding variables; and (3) There is only one possible mechanism by which each SNP is related with the outcome, and that is through the exposure^9^. (More information is available in Fig. 1).

### Data sources and study population

Recently published GWASs evaluated the relationships between SNPs with LST and IBS, thus we used these data to conduct a 2-sample MR investigation (Table 1).

**Table 1:** Characteristics of Data Sources.

Traits	GWAS ID	PMID	Author	Ancestor	Sample size
LST	NA	36,071,172	Wang et al.	European	557,206
IBS	ebi-a-GCST90016564	34,741,163	Eijsbouts et al.	European	486,601
	finn-b-K11_IBS	NA	NA	European	187,028

The LST dataset used summary statistics from a large GWAS and its related meta-analysis studies to find genetic loci related with leisure screen time across different ancestries^10^. Summary statistics of genotype and phenotypic associations are publically available. According to the findings of this research, two forms of LST include sedentary activities such as watching television or using the computer. A questionnaire including the following questions was used to obtain self-reported information for the estimation of LST: “On a typical day, how many hours do you spend watching TV?” “On a typical day, how many hours do you spend using the computer? (Do not include using a computer at work.)” The hours/day of LST were considered to be metrics of exposure (Details in Supplementary Table S1). More than 0.1% of minor allele frequency from the UK Biobank or more than 3 of minor allele count from other studies was as the cutoff for inclusion in the analyses of genotyped and imputed variants. For 19.1–22.5 million SNPs per trait, the results of GWASs that were study-, sex-, and ancestry-specific were meta-analyzed in METAL using the fixed-effects, inverse variance-weighted method. To avoid bias caused by different ethnic groups, we only selected data from European populations for analysis. Throughout the entire genome, 94 significant SNPs (p-values of less than 5 × 10^–8^) linked with LST were found (Supplementary Table S2).

For the IBS dataset, the summary statistics were taken from the most recent released open GWAS database including 53,400 cases and 433,201 population controls of European ancestry^11^.

Considering the possibility of sample overlap from UK Biobank in the exposure and outcome databases can cause a certain degree of bias in the results. We used another open GWAS database (FinnGen consortium) without data from UK Biobank participants including 4,605 cases and 182,423 controls of European ancestry as IBS validation analysis. (finn-b-K11_IBS, https://www.finngen.fi/fi) (Table 1).

The International Classification of Diseases, Tenth Edition (ICD-10) code M13 or a participant's self-report (either prompted, or unprompted on a digestive health questionnaire) of receiving an IBS diagnosis from a physician were used to make the diagnosis of IBS.

### Selection of instrumental variables

In the harmonization section, the effect allele frequency given in the associated GWAS was utilized to detect and exclude all palindromic SNPs in order to calculate the corresponding strand between the two GWAS. A GWAS p-value of < 5 × 10^−8^ and a linkage disequilibrium r^2^ of < 0.001 were used to determine which independent genetic instruments will be used.

The F-statistic was used to approximate the genetic instruments' IV exposure strength. Strong enough to rule out weak instrument bias in the two-sample model if F > 10. F = R^2^ × ((N − 2))/((1 − R^2^)) was used to calculate the F statistics. R^2^ is the total amount of variance that may be explained by the selected SNPs, and N is the sample size. R^2^ was determined via the formula R^2^ = 2 × MAF × (1 − MAF) × Beta^2^^12^.

### Statistical analysis

This investigation utilized a variety of methods due to their varied applicability and statistical efficacy. To calculate the relative importance of potential causes, we relied heavily on the IVW technique. If more than half of the IVs are incorrect, the weighted median estimator (WME) nevertheless produces reliable results^13^. The maximum likelihood method had high accuracy and small standard deviation by maximizing the likelihood function estimated probability distribution parameters^14^. As a newly designed method, RAPS was undertaken to eliminate bias from weak IVs and suitable for usage with both types of pleiotropy^15^. In the IVW model, pleiotropy was evaluated using the intercept test with the MR-Egger regression method, and horizontal pleiotropy was tested with MR-PRESSO by locating and eliminating outliers (NbDistribution = 10,000, SignifThreshold = 0.05)^16^. The results of the study were tested using a leave-one-out sensitivity analysis.

Odds ratios (95% CIs) were utilized to identify the effects of exposure levels greater than or equal to one standard deviation (SD). Considering the LST was the only exposure factor, we therefore estimated study power at an α of 0•05. Statistical significance of MR analysis and the sensitivity estimates was defined as p < 0.05 level with a two-tailed test. All statistical analyses were performed using R software (version 4.2.2) with the "TwoSampleMR" and "MRPRESSO" packages.

We reran the MR after excluding IVs with genome-wide significance (< 5 × 10^–8^) for potential confounding traits such as adiposity, body fat mass, body fat percentage, body mass index and waist-to-hip ratio using the GWAS catalog (https://www.ebi.ac.uk/gwas/) and PhenoScanner V2^17^.

## Results

Application of the selection criteria identified sixty-one SNPs as possible independent IVs related to LST (p < 5 × 10^−8^), none of which were measurably associated with IBS (p > 5 × 10^−8^). The minor instrumental bias can be statistically ignored, as the F-statistic ranged from 90.13 to 341.20 (Supplementary Table S3). We identified a causal relationship between LST and IBS using IVW (OR = 1.120, 95% CI 1.029–1.219, p = 0.009), WME (OR = 1.112, 95% CI 1.000–1.236, p = 0.049), Maximum likelihood (OR = 1.121, 95% CI 1.042–1.207, p = 0.002) and RAPS (OR = 1.124, 95% CI 1.044–1.210, p = 0.002), but not MR-Egger (Table 2 and Supplementary Fig. S1). Horizontal pleiotropy was not detected by the MR-Egger intercept-based test (MR-Egger intercept = 0.009; p = 0.139), indicating an absence of horizontal pleiotropy. Cochrane’s Q value (p-value = 0.026) indicated high heterogeneity in the connection between LST and IBS. However, the MR-PRESSO approach did not identify any outliers, and the causal relationship in the WME remained meaningful (Table 2). The results of the leave-one-out approach proved that the exclusion of SNP had no substantial effect on the results, indicating that the results were reliable (Supplementary Fig. S1).

**Table 2:** Mendelian randomization analysis for assessing the causal effects of LST on IBS.

MR method	OR	95% CI	P-value	N-SNP	Q-value	P-value for heterogeneity^‡^ or pleiotropy^§^
ALL LST-related SNPs				61		
IVW^†^	1.12	1.029–1.219	0.009	61	83.038	0.026^‡^
MR-Egger	0.823	0.545–1.241	0.356	61		0.139^§^
Weighted median	1.112	1–1.236	0.049	61		
Maximum likelihood	1.121	1.042–1.207	0.002	61		
RAPS	1.124	1.044–1.210	0.002	61		
Excluded BMI or adiposity related SNPs				47		
IVW	1.131	1.025–1.248	0.014	47		0.047^‡^
MR-Egger	0.788	0.492–1.261	0.325	47		0.13^§^
Weighted median	1.151	1.020–1.299	0.023	47		
Maximum likelihood	1.134	1.040–1.235	0.004	47		
RAPS	1.136	1.043–1.238	0.004	47		
Validation analysis				48		
IVW	1.287	0.996–1.662	0.054	48		0.773^‡^
MR-Egger	1.723	0.516–5.787	0.38	48		0.627^§^
Weighted median	1.302	0.891–1.903	0.173	48		
Maximum likelihood	1.294	0.998–1.678	0.051	48		
RAPS	1.294	0.879–1.489	0.055	48		

*LST* leisure screen time, *BMI* body mass index, *OR* odds ratio, *CI* confidence intervals, *Q-value* Cochran’s Q statistic, *IVW* inverse variance weighted.

^†^Weights were penalized due to the presence of heterogeneity based on Cochran’s Q statistic.

^‡^P-value for heterogeneity based on Cochran’s Q statistic.

^§^P-value or pleiotropy based on MR-Egger intercept.

**Figure 2:**
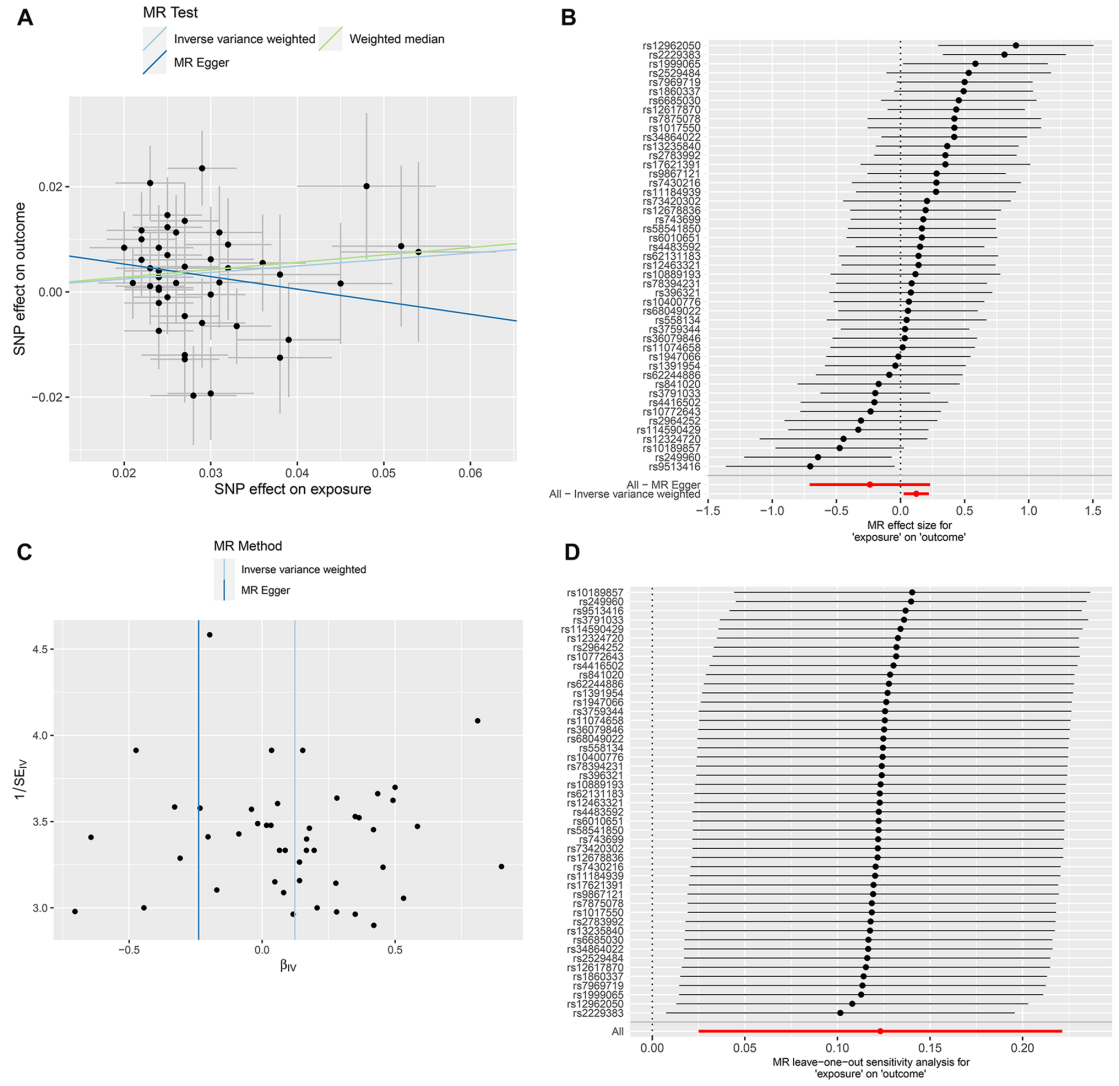
MR results of association between LST and risk of IBS using BMI-unrelated SNPs and shown in scatter plot (**A**), forest plot (**B**), funnel plot (**C**) and leave-one-out sensitivity analysis (**D**).

A higher body mass index (BMI) influences or mediates the causative effects of LST on other disorders. Confounding SNPs must be eliminated to prevent imprecise and potentially biased estimates. We filtered out 20 SNPs associated with body mass index or adiposity (Supplementary Table S2) and used the remaining 47 SNPs to examine the causative effects of LST on irritable bowel syndrome (Supplementary Table S4). For IVW (OR = 1.131, 95% CI 1.025–1.248, p = 0.014), WME (OR = 1.151, 95% CI 1.020–1.299, p = 0.023), Maximum likelihood (OR = 1.134, 95% CI 1.040–1.235, p = 0.004) and RAPS (OR = 1.136, 95% CI 1.043–1.238, p = 0.004) analyses, LST was linked with increased risk of IBS. Using Cochran Q statistics, we found negligible heterogeneity (p = 0.047), while the MR-PRESSO technique found no outliers. No directional pleiotropy was found using the MR-Egger intercept test (p = 0.130). None of the other outliers were found using the leave-one-out sensitivity analysis (Table 2 and Fig. 2).

Furthermore, we used MR analysis on IBS GWAS data from the FinnGen consortium to strengthen the statistical foundation of our findings. Despite only having borderline statistical significance (OR = 1.287, 95% CI 0.996–1.662, p = 0.054), the validation study supported our findings for the connection between LST and IBD, demonstrating identical and consistent effect estimates in IVW and without heterogeneity nor horizontal pleiotropy (Table 2).

## Discussion

It is now widely accepted that leading a sedentary lifestyle is one of the primary risk factors for death caused by non-communicable diseases^18^. These days, people spend more time than they did a decade ago participating in sedentary activities, which may be the result of an increase in screen-related physical activities such as watching television^19^.

Previous research have indicated that IBS is related to physical factors^20–24^. Patients with IBS who engage in more physical activity see a reduction in GI symptoms, most likely as a result of an increase in intestinal transit and the absorptive capacity of the gut^25^. There is also evidence to suggest it can act as a buffer against stress and mental health problems^26^. Decreased physical activity can make a patient more prone to developing functional gastrointestinal disorders because it lowers the body's responsiveness to the immunological changes that are brought on by stress^27^.

Sedentary behavior is often thought of as representing the least active end of the physical activity spectrum. However, new research suggests that important distinctions must be made between sedentary behavior and physical inactivity, since the two have distinct consequences on metabolism, physiology, and prognosis^28,29^.

Available evidence on the detrimental effects of excess LST, a major sedentary behavior, has been observed on intermediate risk factors in previous cross-sectional and prospective studies. In a recent relationship analysis of TV time with adverse health outcomes, 490,966 UK Biobank participants were recruited from 2006 to 2018. This prospective study indicated a substantial relationship between time spent watching television and the risk of death from all causes, including cardiovascular disease (CVD) and malignancies, such as lung, breast, prostate, and colon cancers^6^. In a meta-analysis including 4,784,339 participants, researchers found that each additional two hours spent in television viewing increased the risk of colorectal cancer by 1.07 (95% CI 1.05–1.10)^30^.

As of now, researches into the association between sedentary activity and IBS were scant and yielded conflicting results^20,31^. In a cross-sectional study of medical students who have higher rates of IBS as compared to the general population by previous studies, Vasquez-Rios et al. revealed the sedentary lifestyle was independently associated with IBS (odds ratio: 3.2; 95% CI 1.25–8.20; P = 0.01)^31^. However, in another cross-sectional study, increased physical activity actually worsened the abdominal symptoms of IBS in a state of low mindfulness^20^.

With the current study, Mendelian randomization was used on Europeans to look into the link between LST and IBS risk in the context of sedentary lifestyles. This is the first two-sample MR study investigating the causal relationship between LST and IBS. This method could greatly relieve the lack of reliable evidence that results from the impacts of reverse causation and incomplete adjustment for confounders combined with the relatively small sample sizes of conventional trials. According to the results of our research, having high levels of LST is connected with an increased chance of developing IBS. Our study also strengthens the available evidence on the detrimental effects of excess LST on intermediate risk factors.

When it comes to GI issues, obesity is a major contributor. Evidence from studies conducted on both children and adults shows that those who are overweight are more likely to suffer from IBS. In addition, numerous SNP loci for sedentary behavior, including LST, have previously been associated with adiposity-related features^19,32^, which is consistent with the associations between LST and the risk of overweight/obesity^10,33^. Considering the influence of bias, we eliminated the potentially confounding SNPs and conducted additional research to further verify the causal association between LST and IBS. This was done to avoid precision loss and possibly skewed results using obesity-related SNPs for analysis. All four analysis methods yielded consistent results with statistical significance, which indicated that LST can raise the fractional risk of IBS and that this effect is independent of obesity-related mechanisms. Considering the limitation of sample size, we used another database for validation. Although the trend of the results was consistent with the previous analysis, the statistical significance was only at the critical value. Larger Mendelian randomization studies therefore were encouraged due to the insufficient power to strongly define the relationship between LST and IBS in this study.

This study has some limitations. IBS has been accepted as a disorder of altered bidirectional communication between the gut and brain and has a biopsychosocial etiology. Effective associations have been reported between screen time and sleep problems, depression, and psychological well-being^34^. These factors may affect the pathogenesis of IBS via the brain-gut axis^3,21,35^. Regarding the impact of IBS on sedentary behavior, many studies have found that the proportion of physical inactivity in IBS patients is higher, but few studies have determined that IBS is the cause of higher physical inactivity. However, from the perspective of brain gut axis regulation, which was thought to play a key role in the genesis and maintenance of symptoms in IBS, IBS may further affect physical activity by affecting the nervous biological system. In addition, the majority of study participants were Europeans enrolled in the GWAS biobank; hence, the results cannot accurately represent patients from other areas and races.

In conclusion, this study provides additional evidence that LST is related with a higher incidence of IBS. However, there is a lack of knowledge about the biological pathways through which LST influences IBS, and further research is required to better understand these relationships.

### Supplementary Information

Supplementary Figure S1.

Supplementary Tables.

## Data Availability

The current analysis relied on data from published papers and public databases. They are available at https://www.ebi.ac.uk/gwas/.

## References

[1] Tuck CJ (2022). Changes in signalling from faecal neuroactive metabolites following dietary modulation of IBS pain. Gut.

[2] Ma C (2021). Epidemiologic burden and treatment of chronic symptomatic functional bowel disorders in the United States: A nationwide analysis. Gastroenterology.

[3] Vasant DH (2021). British Society of Gastroenterology guidelines on the management of irritable bowel syndrome. Gut.

[4] Ford AC (2018). American college of gastroenterology monograph on management of irritable Bowel syndrome. Am. J. Gastroenterol..

[5] Lee IM (2012). Effect of physical inactivity on major non-communicable diseases worldwide: An analysis of burden of disease and life expectancy. Lancet (London, England).

[6] Foster HME (2020). Understanding how much TV is too much: A nonlinear analysis of the association between television viewing time and adverse health outcomes. Mayo Clin. Proc..

[7] Li Y (2021). Television viewing time and the risk of colorectal cancer mortality among Japanese population: The JACC study. Cancer Res. Treat..

[8] Ratjen I (2017). Postdiagnostic physical activity, sleep duration, and TV watching and all-cause mortality among long-term colorectal cancer survivors: A prospective cohort study. BMC Cancer.

[9] Skrivankova VW (2021). Strengthening the Reporting of observational studies in epidemiology using mendelian randomization: The STROBE-MR statement. JAMA.

[10] Wang Z (2022). Genome-wide association analyses of physical activity and sedentary behavior provide insights into underlying mechanisms and roles in disease prevention. Nat. Genet..

[11] Eijsbouts C (2021). Genome-wide analysis of 53,400 people with irritable bowel syndrome highlights shared genetic pathways with mood and anxiety disorders. Nat. Genet..

[12] Liu B (2022). Two-sample mendelian randomization analysis investigates causal associations between gut microbial genera and inflammatory bowel disease, and specificity causal associations in ulcerative colitis or Crohn's disease. Front. Immunol..

[13] Hartwig FP, Davies NM, Hemani G, Davey Smith G (2016). Two-sample Mendelian randomization: Avoiding the downsides of a powerful, widely applicable but potentially fallible technique. Int. J. Epidemiol..

[14] Wu F, Huang Y, Hu J, Shao Z (2020). Mendelian randomization study of inflammatory bowel disease and bone mineral density. BMC Med..

[15] Zhao Q, Chen Y, Wang J, Small DS (2019). Powerful three-sample genome-wide design and robust statistical inference in summary-data Mendelian randomization. Int. J. Epidemiol..

[16] Carter AR (2021). Mendelian randomisation for mediation analysis: Current methods and challenges for implementation. Eur. J. Epidemiol..

[17] Kamat MA (2019). PhenoScanner V2: An expanded tool for searching human genotype-phenotype associations. Bioinformatics (Oxford, England).

[18] Lee BY (2017). Modeling the economic and health impact of increasing children's physical activity in the United States. Health Affairs (Project Hope).

[19] Haghjoo P, Siri G, Soleimani E, Farhangi MA, Alesaeidi S (2022). Screen time increases overweight and obesity risk among adolescents: A systematic review and dose-response meta-analysis. BMC Prim. Care.

[20] Koseki T (2023). Impact of mindfulness tendency and physical activity on brain-gut interactions. J. Gastroenterol..

[21] Madva EN (2023). Positive psychological well-being: A novel concept for improving symptoms, quality of life, and health behaviors in irritable bowel syndrome. Neurogastroenterol. Motil..

[22] Groenendijk DW, Witteman BJ, Mulder BC (2022). The experiences of female IBS patients concerning physical activity as treatment modality: A qualitative study. Qual. Health Res..

[23] Nunan D (2022). Physical activity for treatment of irritable bowel syndrome. Cochrane Database Syst. Rev..

[24] Hamaguchi T (2020). The effects of locomotor activity on gastrointestinal symptoms of irritable bowel syndrome among younger people: An observational study. PLoS ONE.

[25] Johannesson E, Simrén M, Strid H, Bajor A, Sadik R (2011). Physical activity improves symptoms in irritable bowel syndrome: A randomized controlled trial. Am. J. Gastroenterol..

[26] Zschucke E, Gaudlitz K, Ströhle A (2013). Exercise and physical activity in mental disorders: Clinical and experimental evidence. J. Prev. Med. Public Health.

[27] Bi L, Triadafilopoulos G (2003). Exercise and gastrointestinal function and disease: An evidence-based review of risks and benefits. Clin. Gastroenterol. Hepatol..

[28] Owen N, Healy GN, Matthews CE, Dunstan DW (2010). Too much sitting: The population health science of sedentary behavior. Exerc. Sport Sci. Rev..

[29] Ekelund U (2016). Does physical activity attenuate, or even eliminate, the detrimental association of sitting time with mortality? A harmonised meta-analysis of data from more than 1 million men and women. Lancet (London, England).

[30] Ma P, Yao Y, Sun W, Dai S, Zhou C (2017). Daily sedentary time and its association with risk for colorectal cancer in adults: A dose-response meta-analysis of prospective cohort studies. Medicine.

[31] Vasquez-Rios G (2019). Stress and a sedentary lifestyle are associated with irritable bowel syndrome in medical students from Peru: A cross-sectional study. Eur. J. Gastroenterol. Hepatol..

[32] Bejarano CM (2021). Physical activity, sedentary time, and diet as mediators of the association between TV time and BMI in youth. Am. J. Health Promot..

[33] Justice AE (2017). Genome-wide meta-analysis of 241,258 adults accounting for smoking behaviour identifies novel loci for obesity traits. Nat. Commun..

[34] Costigan SA, Barnett L, Plotnikoff RC, Lubans DR (2013). The health indicators associated with screen-based sedentary behavior among adolescent girls: A systematic review. J. Adolesc. Health.

[35] Orr WC, Fass R, Sundaram SS, Scheimann AO (2020). The effect of sleep on gastrointestinal functioning in common digestive diseases. Lancet Gastroenterol. Hepatol..

