# Supplementary material for: The causal effects of leisure screen time on irritable bowel syndrome risk from a Mendelian randomization study
Source: Sci Rep. 2023 Aug 14;13:13216. doi: 10.1038/s41598-023-40153-1 (PMC10425325; doi:10.1038/s41598-023-40153-1)

## Supplemental figure legends

MR results of association between LST and risk of IBS using all SNPs and shown in scatter plot (A), funnel plot (B) and leave-one-out sensitivity analysis (C).

## Supplemental figure 1

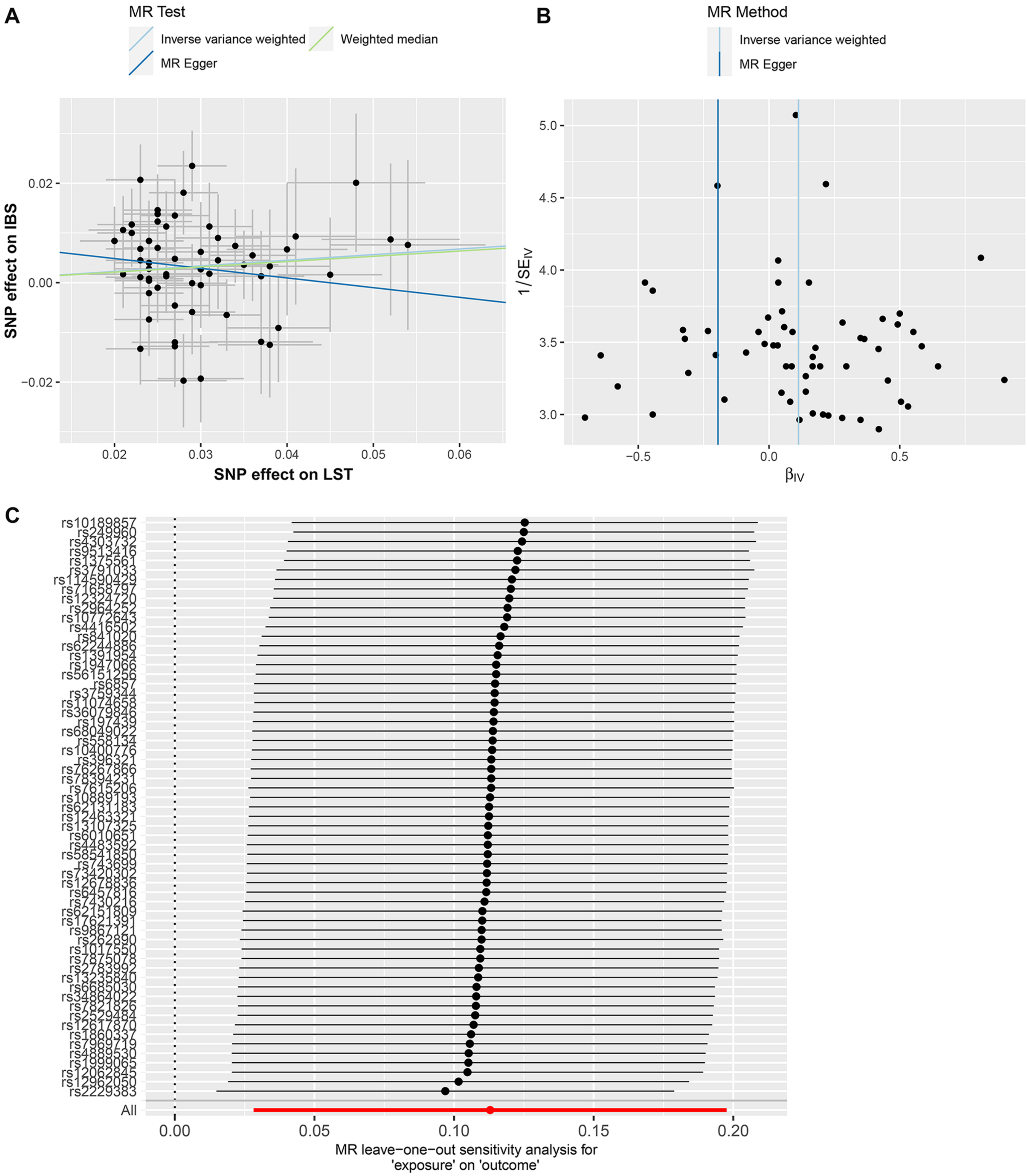

Supplement: Supplementary file 1 — Supplementary Figure S1. [file 41598_2023_40153_MOESM1_ESM.pdf]
